# Albumin-Bilirubin (ALBI) Score and Systemic Immune-Inflammation Indexes Used As Pretreatment Outcome Predictors in Patients With Pancreatic Ductal Adenocarcinoma Undergoing Robotic or Open Whipple Procedures: A Logistic Regression Analysis

**DOI:** 10.7759/cureus.50949

**Published:** 2023-12-22

**Authors:** Alejandro Mejia, Elaina Vivian, Jimmy Shah, Juan Carlos Barrera Gutierrez

**Affiliations:** 1 Surgery, The Liver Institute, Methodist Dallas Medical Center, Dallas, USA; 2 Performance Improvement, Methodist Dallas Medical Center, Dallas, USA

**Keywords:** logistic regression analysis, systemic immune-inflammation index, albumin-bilirubin score, prognostication, pancreatoduodenectomy, pancreatic ductal adenocarcinoma

## Abstract

Background

Pancreatic ductal adenocarcinoma (PDAC) is the most common type of pancreatic cancer (PC) in the United States. In patients with resectable PC, identification of pretreatment biomarkers before surgery can help in the decision-making process by weighing the benefits of neo-adjuvant therapy, surgical procedure, and adjuvant therapy. The purpose of this study was to determine if the albumin-bilirubin (ALBI) score and immune-inflammatory marker levels can be used in combination as pretreatment predictors of mortality risk in patients undergoing the Whipple procedure (alternatively, pancreatoduodenectomy (PD)) for PDAC.

Methods

This retrospective study included 115 patients with PDAC who underwent open or robotic Whipple procedures between January 2013 and December 2022 at a single tertiary medical center. Logistic regression analysis was used to find the association between predictors and mortality. Machine learning algorithms were used to calculate the performance of the different models.

Results

Bivariate analysis showed that the variables “sex” and “body mass index (BMI)” had a potential association with mortality, although statistical significance was not achieved for sex (p = 0.07). Patients with BMIs >25 kg/m^2^ had a higher risk of mortality compared to patients with BMIs ≤24.9 kg/m^2 ^(odds ratio (OR) = 2.2, 95% CI = 1.03-4.8, p = 0.04). Higher (more positive) ALBI scores (>-2.24) were also associated with increased mortality risk (OR = 4.6, 95% CI = 2-10.5, p = 0.0003). When the cutoff values of the inflammatory markers were used to categorize these variables, values greater than the cutoff values were associated with an increased risk of mortality. In the multivariate logistic regression model, an ALBI score >-2.24 (OR = 4.3, 95% CI = 1.8-10.3, p = 0.0008), neutrophil-to-lymphocyte ratio (NLR) >3.5 (OR = 3.3, 95% CI = 1.4-7.9, p = 0.007), and being a woman (OR = 2.6, 95% CI = 1.1-6.4, p = 0.03) remained influential predictors of increased mortality (c value = 0.77).

Conclusion

The ALBI score and the NLR are easily accessible markers; their use, combined with a patient’s sex, can provide useful pre-surgical information regarding mortality risk after PD. This can aid in treatment planning as well as expedite decisions about the type of Whipple procedure, adjuvant therapy, and surveillance, which can subsequently improve a patient’s outcomes and survival.

## Introduction

Pancreatic ductal adenocarcinoma (PDAC) is the most common type of pancreatic cancer (PC) in the United States. It is the fourth leading cause of cancer death [1], with a five-year survival rate of 11.5% [2]. Because of the difficulty in obtaining an early diagnosis and the limited response to treatments, PDAC is extremely lethal. There are a few pretreatment biomarkers of prognostic outcomes after PC treatment, including carbohydrate antigen 19-9 (CA 19-9). Lower levels of serum CA 19-9 (<37 U/mL) have been associated with longer median survival times (32-36 months) in PC patients. Conversely, higher levels of serum CA 19-9 (>37 U/ml) were associated with shorter median survival (12-15 months) [3]. The role of CA 19-9 in PC diagnosis, staging, determining resectability, response to chemotherapy, and prognosis has not been definitively defined [3]. In resectable PDAC, an optimal CA 19-9 cutoff value of 338.45 U/ml is a predictor of poor prognosis [4]. However, a limitation of using CA 19-9 to predict PDAC prognosis is the variability in the optimal cutoff values reported in different studies. Also, the poor sensitivity of CA 19-9 in the diagnosis of PC (namely, PDAC) increases the probability of type 1 errors [3,4].

Markers of systemic inflammation, such as the systemic immune-inflammation index (SII), the neutrophil-to-lymphocyte ratio (NLR), and the platelet-to-lymphocyte ratio (PLR), have been used as predictors of advanced cancer [5]. A meta-analysis found that high NLR indicated poor overall survival (OS) prognosis in PC patients [6]. Other studies showed that NLR was an independent predictor of progression-free survival and OS in PCs [7].

The albumin-bilirubin score (ALBI score) has been used to assess the severity of liver disease in patients with hepatocellular carcinoma, chronic liver disease [8], colorectal liver metastases [9], and recently in PC [10, 11]. Yagyu et al. demonstrated that the five-year OS rate in resected PC patients with high ALBI scores and high (>35 U/ml) CA 19-9 levels was 13.8%, whereas patients with low ALBI (more negative) scores and low CA 19-9 levels had a survival rate of 43.3% [10]. Furthermore, high ALBI scores are associated with worse OS and progression-free survival in PC patients with liver metastasis treated with chemotherapy [12].

In patients with resectable PC, identification of pretreatment biomarkers before surgery can help in the decision-making process by weighing the benefits of neo-adjuvant therapy, surgical procedure, and adjuvant therapy. Furthermore, identifying patients at high risk of mortality before surgery allows physicians to make informed decisions regarding early adjuvant therapy initiation and close surveillance. This approach improves adherence to neo-adjuvant and adjuvant chemotherapy, especially in those at high risk of mortality or early recurrences. The purpose of this study is to determine if ALBI scores and inflammatory markers can be used in combination as pretreatment predictors of mortality risk in patients undergoing Whipple procedures, also called proximal pancreaticoduodenectomy (PD). This surgical procedure involves the removal of the head of the pancreas, duodenum, gallbladder, and bile duct.

## Materials and methods

Study cohort

This single-center retrospective study included 115 patients with PDAC who underwent either an open PD (OPD) (39 procedures) or robotic PD (RPD) (76 procedures) between January 2013, the year when RPD was standardized as an approach to resectable PDAC in our institution, and December 2022. Nineteen patients received neo-adjuvant therapy for a four-month duration; however, the compliance rate was less than 50%. Neo-adjuvant therapy using the FOLFIRINOX (irinotecan, oxaliplatin, and infusional fluorouracil) protocol was offered to those patients with borderline resectable PDAC with the purpose of improving tumor resectability and overall survival. Eighty-seven patients received adjuvant therapy based on a six-month modified FOLFIRINOX regimen, which was initiated within 10 weeks after surgery unless contraindicated. The included patients were >18 years old, with histological confirmation of PDAC without distant metastasis and a performance status of 0 to two. Patients with obstructive jaundice (with total bilirubin <15 mg/dL) received endoscopic retrograde cholangiopancreatography with stent placement as part of routine care to improve the patient's nutritional status and quality of life. The median (min-max) follow-up was 19.7 (0-82) months. The study was approved by the WIRB-Copernicus Group (WCG) Institutional Review Board (Puyallup, WA) and was found to meet the requirements for a waiver of consent under 45 CFR 46 116(f) (2018 requirements). All the surgical procedures were recommended as per the standard of care for pancreatic cancer. The specific protocol for robotics and open cases was followed and has been described in the literature previously [13].

Predictor variables

Available data included basic demographic characteristics, such as age, sex, race/ethnicity, body mass index (BMI), and comorbidities (e.g., hypertension, diabetes, coronary vascular disease, etc.). Baseline laboratory data was measured the week or day before the surgery, including hemoglobin, bilirubin, albumin, and absolute platelet, neutrophil, and lymphocyte counts. Additionally, CA 19-9 levels were collected. Symptoms of jaundice, comorbidity profile, the Eastern Cooperative Oncology Group (ECOG) performance status scale, tomographic tumor size, and receipt of neo-adjuvant therapy were included.

Calculation of predictor variables

The ALBI score was calculated from two variables (albumin and total bilirubin) with the following formula: ALBI score = (-0.085 x albumin (g/dL)) + (0.66 x log10 total bilirubin (mg/dL)) [8]. The SII was defined as follows: SII = (platelet x neutrophil)/lymphocyte. The NLR from the absolute blood count was calculated as NLR = neutrophil/lymphocyte, and the PLR was calculated as PLR = platelet/lymphocyte [14].

Response variables

The primary endpoint was mortality during the follow-up period after the Whipple procedure. Follow-up time (measured in months) was defined as the time from the date of the Whipple procedure to the date of the patient’s death, the date of the last follow-up, or the date of the end of the study.

Statistical analysis

Statistical analyses were carried out using Statistical Analysis System OnDemand for Academics (RRID: SCR_008567, SAS Institute Inc., Cary, NC) and Python version 3.9 (RRID: SCR_006903, Python Software Foundation, Wilmington, DE). Baseline characteristics were described with means and standard deviations (SD), or median and minimum to maximum (min-max) values, according to the normal or non-normal distribution of continuous variables, respectively. T-tests or Wilcoxon rank tests were used to compare these continuous variables based on the normal or non-normal distribution, respectively. Categorical variables were summarized with percentages and compared with Pearson chi-square tests. The cutoff values of the biomarkers were established with receiver operating characteristic (ROC) curve analysis and Youden’s index, which provided the highest sensitivity and specificity. The correlations between the pre-surgical laboratories, ALBI scores, inflammatory markers, and mortality were tested with Pearson’s correlation coefficient. Logistic regression was used to establish the association between pretreatment predictors and mortality. Manual step-backward variable selection was used to build a multivariable logistic regression model including variables with a p <0.2 and keeping in the model those variables with a p <0.05. Machine learning algorithms were used to calculate the performance of the different models using training and testing datasets at a ratio of 0.7 to 0.3, respectively. Machine learning facilitates model evaluation performance by utilizing various metrics and techniques to assess and sample the data. The performance metrics included accuracy, sensitivity, precision, and F1 score. The F1 score evaluates the model; the greater the F1 score, the higher the chances of minimizing false negatives and false positives. Subgroup analyses were done for OPD and RPD. Bagging (bootstrapping aggregation) was utilized to improve the overall performance and generalization of the predictive model. With the bagging technique, multiple instances of the same base model were trained on different subsets of the training data, employing a process of random sampling with replacement. The predictions of these individual models were combined through averaging, aiming to reduce overfitting and enhance model stability. Missing ECOG status values were calculated using the mode value according to the type of surgery. Finally, Kaplan-Meier curves were calculated for the pre-surgical biomarkers associated with mortality, and log-rank tests were used to test the differences between groups. P-values <0.05 were considered statistically significant.

## Results

Patient characteristics

A total of 115 patients underwent Whipple procedures between 2013 and 2022 (Table 1).

**Table 1:** Descriptive baseline characteristics of survivors and deceased patients after pancreaticoduodenectomy BMI: body mass index; ECOG: Eastern Cooperative Oncology Group scale; CA 19-9: carbohydrate antigen 19-9; ALBI: albumin-bilirubin; SII: systemic immune-inflammatory index; NLR: neutrophil-to-lymphocyte ratio; PLR: platelet-to-lymphocyte ratio *: non-parametric test

	Total (N=115)	Survivors (N=49)	Deceased (N=66)	
Variable	n (%)	n (%)	n (%)	p-value
Age, years, mean (SD)	66 (10.1)	67.3 (10.6)	65.6 (9.7)	0.4
Sex				
Female	46 (40)	15 (31)	31 (47)	0.08
Male	69 (60)	34 (69)	35 (53)	
Race/ethnicity				
Black	28 (24)	12 (25)	16 (24)	0.98
White	72 (63)	31 (63)	41 (62)	
Hispanic	15 (13)	6 (12)	9 (14)	
BMI (kg/m^2^)				
≤24.9	44 (38)	24 (49)	20 (30)	0.12
>25 to ≤29.9	38 (33)	14 (29)	24 (36)	
≥30	33 (29)	11 (22)	22 (33)	
Number of comorbidities				
0-1	54 (47)	20 (41)	34 (52)	0.26
2 or more	61 (53)	29 (59)	32 (48)	
ECOG status				
0-1	70 (61)	36 (75)	34 (52)	0.01
2	22 (19)	4 (8)	18 (27)	
Missing	22 (19)	8 (17)	14 (21)	
Tumor size (cm)				
≤2	10 (9)	4 (8)	6 (9)	0.7
>2 - 4	72 (63)	29 (59)	43 (65)	
>4	33 (29)	16 (33)	17 (26)	
Jaundice				
Yes	65 (57)	27 (55)	38 (58)	0.8
No	50 (43)	22 (45)	28 (42)	
Neo-adjuvant therapy				
Yes	19 (17)	7 (14)	12 (18)	0.6
No	96 (83)	42 (86)	54 (82)	
Type of procedure				
Robotic	76 (66)	36 (73)	40 (61)	0.15
Open	39 (34)	13 (27)	26 (39)	
Baseline laboratories				
Hemoglobin, g/dL, mean (SD)	12 (1.7)	12.3 (1.6)	11.8 (1.7)	0.15
Bilirubin, mg/dL, median (min-max)	1.4 (0.2-25)	1.1 (0.2-14.2)	1.6 (0.3-25)	0.01
Albumin, g/dL, mean (SD)	3.7 (0.5)	3.9 (0.5)	3.5 (0.5)	0.0003
Lymphocytes x 10^3^cells/µL, mean (SD)	1.5 (0.6)	1.6 (0.7)	1.4 (0.4)	0.16
Neutrophils x 10^3^cells/µL, mean (SD)	5.3 (2.5)	4.7 (2.5)	5.7 (2.4)	0.03
Platelets x 10^3^cells/µL, mean (SD)	234 (71)	233 (69)	235 (73)	0.9
CA 19-9*, U/ml, median (min-max)	208 (1.4-11200)	171 (1.4-11200)	216 (1.4_5420)	0.7
ALBI score, mean (SD)	-2.1 (0.6)	-2.4 (0.5)	-1.9 (0.7)	< .0001
SII* median (min-max)	738 (191-6799)	609 (208-6799)	909 (191-3408)	0.01
NLR, median (min-max)	3.3 (1-278)	2.6 (1.1-28)	3.9 (1-14.5)	0.003
PLR*, median (min-max)	149 (69-613)	135 (69-613)	162 (75-588)	0.2
Follow-up, days, median (min-max)	19.7 (0-82)	26 (1-82)	15 (0-55)	0.0009

The mean (SD) age of the patients was 66 (±10.1) years. There was a greater proportion of men (60%) than women (40%), and 63% were non-Hispanic White patients. Baseline laboratories showed a mean (SD) hemoglobin level of 12 (±1.7) mg/dL, albumin of 3.7 (±0.5) g/dL, an absolute lymphocyte count of 1.5 (±0.6) x 103 lymphocytes/µL, a neutrophil count of 5.3 (±2.5) x 103 neutrophils/µL, and a platelet count of 234 (±71) x 103 platelets/µL. The median (min-max) total bilirubin and CA 19-9 levels were 1.4 (0.2-25) mg/dL and 208 (1.4-11.2) U/mL, respectively. The mean (SD) ALBI score was -2.1 (±0.6), and the median (min-max) SII, NLR, and PLR were 738 (191-6799) x 109 cells/L, 3.3 (1-27.8), and 149 (69-613), respectively. Robotic PD accounted for 66% (76/115) of the procedures. The overall mortality rate after the Whipple procedures was 57% (66/115) during a median (min-max) follow-up period of 19.7 (0-82) months.

Patients who died during the follow-up period exhibited higher values of several key variables compared to patients who survived, including an ECOG status of two (27% vs. 8%, p = 0.01), median (min-max) bilirubin levels 1.6 (0.3-25) vs. 1.1 (0.2-14.2 mg/dL, p = 0.01), and mean (SD) ALBI scores (-1.9 (0.7) vs. -2.4 (0.5), p <0.0001). In contrast, deceased patients displayed lower mean (SD) albumin concentrations (3.5 (0.5) vs. 3.9 (0.5) g/dL, p = 0.0003) compared to survivors. Deceased patients also demonstrated higher median (min-max) NLR (3.9 (1-14.5) vs. 2.6 (1.1-28), p = 0.003) and SII values (909 (191-3408) vs. 609 (208-6799) x 109 cells/L, p = 0.01) compared to survivors, indicating more intense systemic inflammatory and immune responses in the non-surviving cohort.

Analysis of the ROC curve, cutoff points, and correlation between pre-surgical biomarkers

The cutoff points of several potential pre-surgical markers were determined (Table 2).

**Table 2:** Cutoff values of pre-surgical markers used to predict mortality in pancreatic ductal adenocarcinoma: C-statistic, sensitivity, specificity, Youden index, and odds ratios for different cutoff values OR: odds ratio; ALBI: albumin-bilirubin; SII: systemic immune-inflammatory index; NLR: neutrophil-to-lymphocyte ratio; PLR: platelet-to-lymphocyte ratio

Variable	Cutoff	C-statistic	Sensitivity	Specificity	Youden index	OR	p-value
Hemoglobin	11.7	0.59	0.71	0.48	0.2443	2.4	0.03
ALBI score	-2.24	0.715	0.69	0.68	0.4007	4.9	0.0001
SII	779	0.64	0.69	0.59	0.2847	3.3	0.003
NLR	3.5	0.66	0.73	0.59	0.346	4	0.0007
PLR	145	0.57	0.59	0.62	0.285	2.4	0.002

The cutoff point for the ALBI score was -2.24. This value showed the highest C-statistic (0.715), Youden index (0.4007), and odds ratio (OR) (4.9, p = 0.0001) values. Among the markers of inflammation, NLR was selected as the best marker. With a cutoff point of 3.5, NLR had the highest C-statistic (0.66), Youden index (0.346), and OR (4, p = 0.007) values. These cutoff points were used to categorize the same variables into two groups: high scores or low scores. The cutoff value for CA 19-9 was not used given the high percentage of missing values and low statistical significance.

Logistic regression to establish the association of pretreatment markers and mortality risk after a Whipple procedure

The relationship between patient characteristics and pre-surgical biomarkers and mortality risk after a Whipple procedure was assessed (Table 3).

**Table 3:** Logistic regression analysis to establish the association of pretreatment markers and patients’ characteristics with mortality after a Whipple procedure OR: odds ratio; CI: confidence interval; BMI: body mass index; ECOG: Eastern Cooperative Oncology Group scale; CA 19-9: carbohydrate antigen 19-9; ALBI: albumin-bilirubin; SII: systemic immune-inflammatory index; NLR: neutrophil-to-lymphocyte ratio; PLR: platelet-to-lymphocyte ratio *: non-parametric test

Variable	OR	95% CI	p-value
Age	0.99	0.95-1.02	0.4
Sex			
Female	2	0.9-4.4	0.07
Male	Ref		
Race/ethnicity			
Black	Ref		
White	0.99	0.4-2.4	0.99
Hispanic	1.1	0.3-4	0.9
BMI, kg/m^2^			
≤24.9	Ref		
>25	2.2	1.03-4.8	0.04
Number of comorbidities			
0-1	Ref		
≥2	0.6	0.3-1.4	0.3
ECOG status			
0-1	Ref		
2	4.2	1.3-13	0.01
Tumor size, cm			
≤2	Ref		
>2 - 4	0.99	0.2-3.8	0.99
>4	0.7	0.2-3	0.6
Jaundice			
Yes	1.1	0.5-2.3	0.8
No	Ref		
Neo-adjuvant therapy			
Yes	Ref		
No	0.8	0.3-2	0.6
Type of procedure			
Robotic	Ref		
Open	1.8	0.8-4	0.15
Baseline laboratories			
Hemoglobin	0.843	0.7-1.1	0.15
Bilirubin	1.13	1-1.3	0.14
Albumin	0.2	0.1-0.54	0.0008
Lymphocyte	0.6	0.33-1.2	0.15
Neutrophil	1.2	1-1.4	0.03
Platelets	1	1-1.006	0.9
CA 19-9*	1	1-1	0.4
ALBI score	4.6	2-10.5	0.0003
SII*	1	1-1	0.3
NLR	1.1	0.96-1.2	0.2
PLR	1	1-1	0.5
Continuous variables according to cutoff value			
Hemoglobin group			
≤11.7 g/dL (low)	2.4	1.1-5.2	0.03
>-11.7 g/dL (high)	Ref		
ALBI group			
≤-2.24 (low)	Ref		
>-2.24 (high)	4.9	2.2-10.8	0.0001
NLR group			
≤3.5 (low)	Ref		
>3.5 (high)	4	1.8-9	0.0007
SII group			
≤779 (low)	Ref		
>779 (high)	3.3	1.5-7.1	0.003
PLR group			
≤145 (low)	Ref		
>145 (high)	2.4	1.1-5.1	0.02

Bivariate logistic regression analysis, including all 115 patients, showed a BMI >25 kg/m^2^ was associated with an increased risk of mortality compared to a BMI ≤24.9 kg/m^2^ (OR = 2.2, 95% CI = 1.03-4.8, p = 0.04). Also, sex had a potential, although not statistically significant, association with increased risk of mortality, with females showing an increased risk of mortality compared to males (OR = 2, 95% CI = 0.9-4.4, p = 0.07).

Among the laboratory values analyzed, albumin and neutrophil levels exhibited associations with an increased risk of mortality. High albumin levels showed a strong association with decreased mortality risk (OR = 0.2, 95% CI = 0.1-0.54, p = 0.0008); for each gram increase of albumin, mortality decreased by 80%. Higher neutrophil levels were associated with a higher risk of mortality (OR = 1.2, 95% CI = 1-1.4, p = 0.03).

The ALBI score, hemoglobin, NLR, SII, and PLR were analyzed as continuous variables. Higher ALBI scores were associated with an increased risk of mortality (OR = 4.6, 95% CI = 2-10.5, p = 0.0003). Moreover, hemoglobin levels <11.7 g/dL were associated with a higher risk of mortality than hemoglobin levels ≥11.7 g/dL (OR = 2.4, 95% CI = 1.1-5.2, p = 0.03). The categorical variables ALBI group, NLR group, SII group, and PLR group were also assessed for associations with increased risk of mortality. The ALBI values >-2.24 were associated with an increased risk of mortality compared to values ≤-2.24 (OR = 4.9, 95% CI = 2.2-10.8, p = 0.0001). Higher cutoff values for NLR (>3.5; OR = 4, 95% CI = 1.8-9, p = 0.0007), SII group (>779 x 109 cells/L; OR = 3.3, 95% CI = 1.5-7.1, p = 0.003), and PLR group (>145; OR = 2.4, 95% CI = 1.1-5.1, p = 0.02) were also associated with increased mortality risk. Together, these findings suggest that certain clinical characteristics and pre-surgical biomarkers are potential predictors of mortality following Whipple procedures.

Correlation analysis

**Figure 1:**
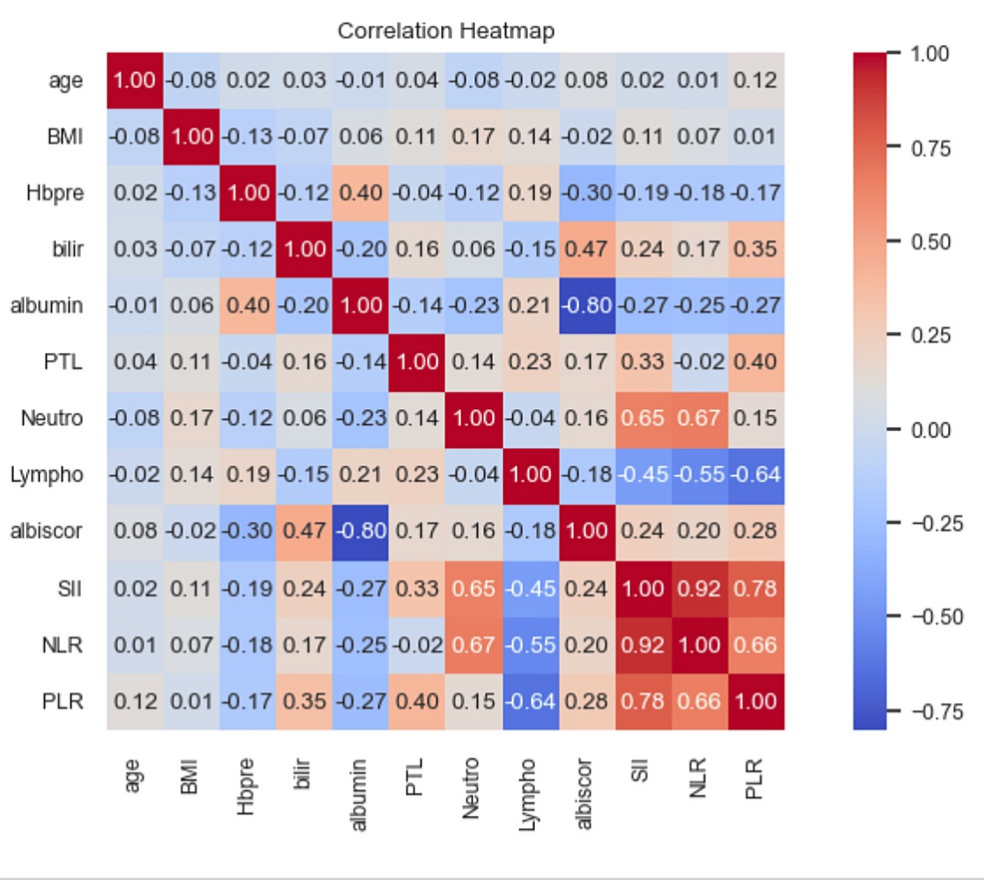
Correlation heat map to examine the interrelationships between continuous variables BMI: body mass index; Hbpre: hemoglobin pre-surgery; bilir: bilirubin; PTL: platelets; Neutro: neutrophils; Lympho: lymphocytes; SII: systemic immune-inflammatory index; NLR: neutrophil-to-lymphocyte ratio; PLR: platelet-to-lymphocyte ratio

A correlation heatmap was utilized to examine the interrelationships among the variables under investigation (Figure 1).

High correlations (r > 0.7) were observed among NLR, SII, and PLR. Additionally, the ALBI score was found to exhibit a high correlation with albumin and a moderate correlation (r = 0.47) with bilirubin. Hemoglobin displayed a mild inverse correlation (r = 0.3) with the ALBI score. To address concerns regarding potential multicollinearity among these highly correlated variables and ensure the integrity of the multivariable logistic model, a decision was made to include NLR and the ALBI score as the selected variables. By adopting this approach, the study aims to mitigate potential confounding effects and ascertain the independent contributions of each variable to the model’s predictive capacity.

Multivariate logistic regression analysis

The multivariate logistic regression analysis in this study (n = 115) incorporated several key variables, including sex, BMI, ECOG status, hemoglobin level, ALBI group, and NLR groups (Table 4).

**Table 4:** Pre-procedural predictors and mortality using a multivariable logistic regression model OR: odds ratio; CI: confidence interval; ALBI: albumin-bilirubin; NLR: neutrophil-to-lymphocyte ratio

Variable	OR	95% CI	p-value
ALBI group			
≤-2.24 (low)	Ref		
>-2.24 (high)	4.3	1.8-10.3	0.0008
NLR group			
≤3.5 (low)	Ref		
>3.5 (high)	3.3	1.4-7.9	0.007
Sex			
Female	2.6	1.1-6.4	0.03
Male	Ref		

After conducting the necessary adjustments, women had a 2.6-fold increased risk of mortality compared with men (OR = 2.6, 95% CI = 1.1-6.4, p =0.03). Higher ALBI scores (>-2.24) were associated with an increased risk of mortality compared to lower ALBI scores (≤-2.24) (OR = 4.3, 95% CI = 1.8-10.3, p = 0.0008). Similarly, higher NLR (>3.5) demonstrated a significant association with increased risk of mortality compared to lower NLR (≤3.5) (OR = 3.3, 95% CI = 1.4-7.9, p = 0.007). These findings suggest that the ALBI group, the NLR group, and sex are the most prominent factors affecting the risk of mortality in this context

Performance of the models

**Figure 2:**
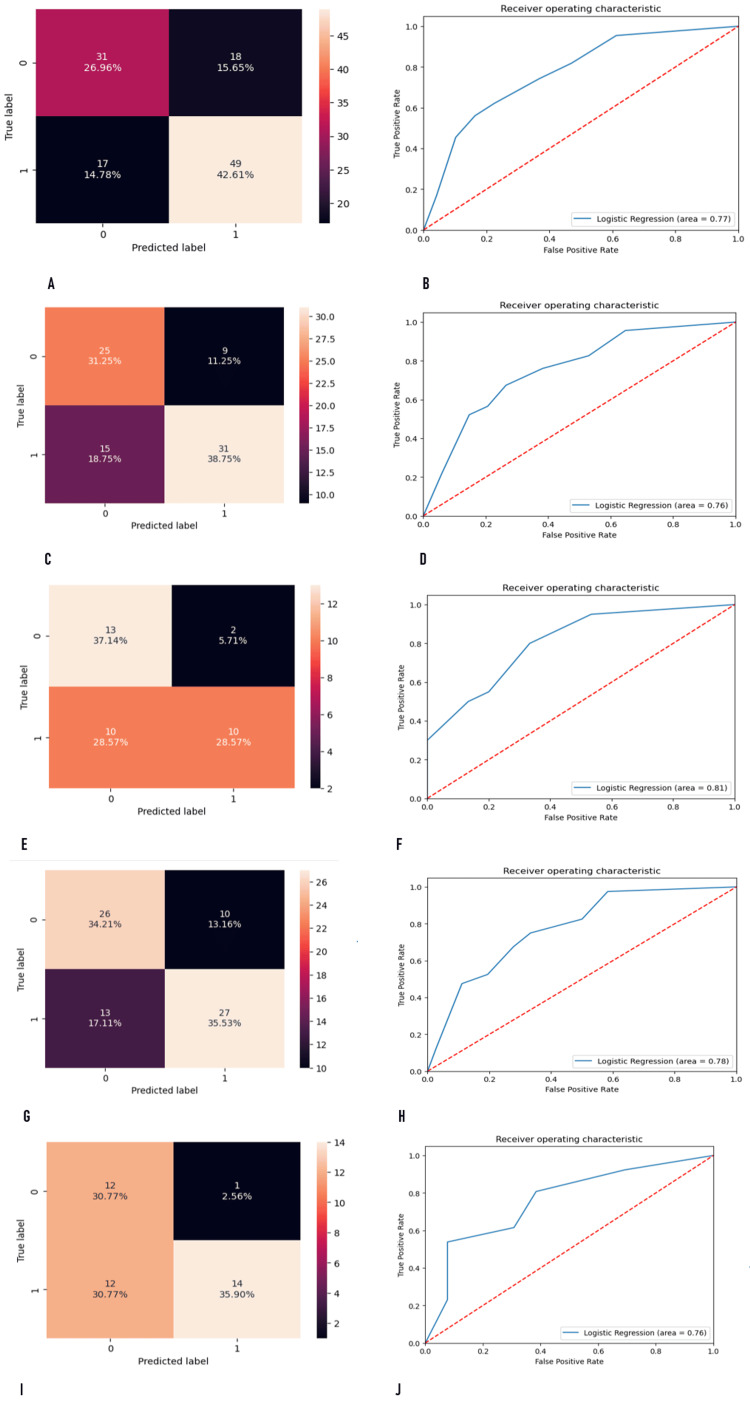
Performance of the different models in the study (A) Contingency table illustrating the four possible outcomes of a binary classifier model in the total sample. (B) Receiver operating characteristic analysis with a true positive rate against a false positive rate in the total sample. (C) The contingency table illustrating the four possible outcomes of a binary classifier model in the training data sample. (D) Receiver operating characteristic analysis with a true positive rate against a false positive rate in the training data sample. (E) The contingency table illustrating the four possible outcomes of a binary classifier model in the testing data sample. (F) Receiver operating characteristic analysis with a true positive rate against a false positive rate in the testing data sample. (G) The contingency table illustrating the four possible outcomes of a binary classifier model in the robotic surgery sample. (H) Receiver operating characteristic analysis with a true positive rate against a false positive rate in the robotic surgery sample. (I) The contingency table illustrating the four possible outcomes of a binary classifier model in the open surgery sample. (J) Receiver operating characteristic analysis with a true positive rate against a false positive rate in the open surgery sample.

The performance of the models to predict mortality risk after PD was evaluated, with a particular focus on the area under the ROC curve (AUC) and sensitivity (Table 5, Figure 2).

**Table 5:** Performance metrics of the models predicting mortality after pancreaticoduodenectomy AUC: area under the curve; F1: F1 score; PD: pancreaticoduodenectomy

Models & data	AUC	Accuracy	Sensitivity	Precision	F1
Overall model	0.77	0.7	0.74	0.73	0.74
Training data	0.76	0.7	0.67	0.78	0.72
Testing data	0.81	0.66	0.5	0.83	0.63
Robotic PD	0.78	0.7	0.68	0.73	0.7
Open PD	0.76	0.67	0.54	0.93	0.68
Bagging logistic regression					
Training data	0.74	0.76	0.86	0.75	0.8
Testing data	0.76	0.77	0.85	0.77	0.79
Robotic PD	0.77	0.78	0.88	0.74	0.8
Open PD	0.69	0.69	0.69	0.81	0.75

In the overall model, the AUC was 77%, indicating reasonably good discrimination ability. The model achieved an accuracy of 70%, but the sensitivity was even higher at 74%, suggesting that the model performs well in correctly identifying patients at risk of mortality. The precision and F1 score were also at acceptable levels (0.73 and 0.74, respectively). However, when the model was evaluated separately on the training and testing data, some differences were observed. The AUC for the training data was 76%, indicating good discrimination ability, like the overall model. However, the sensitivity dropped to 67% for the training data, suggesting that the model's ability to identify patients at risk of mortality was slightly compromised in this dataset. The testing data showed an even higher AUC of 81%, indicating excellent discrimination ability. However, the sensitivity of the testing data was 50%, considerably lower than that of the training data and overall model. Similar trends were observed when examining the performance of the models on RPD and OPD separately. Both types of surgery demonstrated good AUC values (78% and 76% for RPD and OPD, respectively); however, the sensitivity for OPD was lower (54%) than RPD (68%).

To mitigate the impact of a small sample size and help reduce the variance of the model on model performance, a bagging logistic regression model was used. After bagging, the AUC and sensitivity for both the training (AUC = 74%, sensitivity = 86%) and testing (AUC = 76%, sensitivity = 85%) data improved (Table 5). Similarly, both RPD (AUC = 77%, sensitivity = 88%) and OPD (AUC = 69%, sensitivity = 69%) showed higher AUC and sensitivity values when using the bagging model.

Kaplan-Meier curves for the combination of ALBI and NLR groups

**Figure 3:**
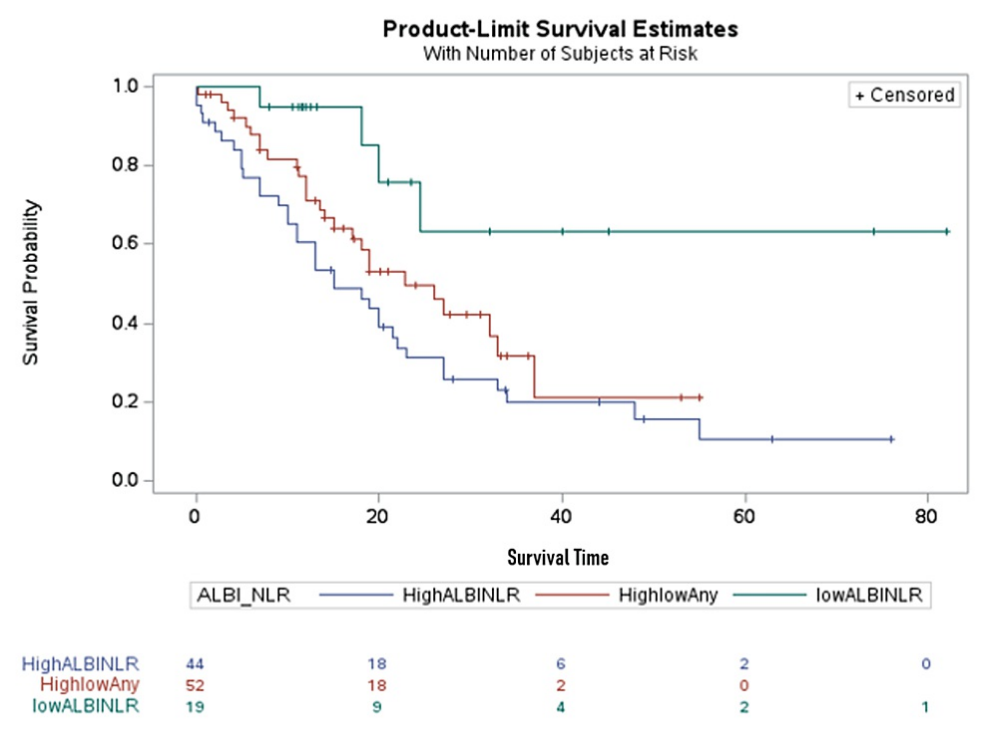
Kaplan-Meier curves for the ALBI score and NLR groups (log-rank test: 0.0061) Blue curve: HighALBINLR = patients with a high ALBI score (>-2.24) and a high NLR ratio (>3.5). Red curve: HighlowAny = patients with high ALBI and low NLR, or low ALBI and high NLR. Green curve: lowALBINLR = low ALBI score (<-2.24) and low NLR (<3.5) ALBI: albumin-bilirubin; NLR: neutrophil-to-lymphocyte ratio

Patients with an ALBI score ≥-2.24 and NLR ≥3.5 were grouped as the “high ALBI and NLR” group, while patients with an ALBI score <-2.24 and NLR <3.5 were grouped as the “low ALBI and low NLR” group. Patients with a combination of high or low ALBI or NLR were grouped as the “high or low any group." The median survival time was >25 months (95% CI = 22-∞) in the “low ALBI and low NLR” group, 22.7 months (95% CI = 15-33) in the “high or low any group," and 15 months (95% CI = 10-22) in the “high ALBI and NLR” group (Figure 3).

The log-rank test yielded a value of 0.0061.

## Discussion

The present study investigated the utility of the ALBI score and NLR as easily accessible markers for predicting mortality after a Whipple procedure. Our findings revealed that the ALBI group (cutoff value: >-2.24), the NLR group (cutoff value: >3.5), and sex were the most prominent factors in predicting mortality during the follow-up period. The use of these biomarkers can offer valuable pre-surgical information, aid in treatment planning, and potentially expedite the decision about the type of Whipple procedure and the use of adjuvant therapy post-surgery. Patients with higher levels of biomarkers will require close surveillance to identify early local recurrence or metastatic disease.

Several factors have been identified to significantly influence postoperative outcomes, with preoperative factors serving as crucial indicators of early mortality. Studies identified age, C-reactive protein levels, CA 19-9, diabetes mellitus, and active smoking as independent risk factors for early mortality [15]. Additionally, preoperative biliary stent placement, preoperative cholangitis, ECOG status, and the presence of frailty syndrome were found to be potential determinants of postoperative prognosis [16]. It is also imperative to recognize the impact of postoperative markers that have demonstrated significant associations with mortality. Tumor size, tumor grade, residual tumor or margin involvement, and the presence of lymph node metastases were identified as critical determinants influencing patient outcomes after pancreatic resection [17, 18]. Furthermore, postoperative variables, such as the International Study Group of Pancreatic Surgery-defined complications, and the utilization of adjuvant therapy, exert notable impacts on postoperative mortality rates [18]. Despite the relevance of these postoperative factors, the current study focused on identifying easily accessible and non-invasive preoperative markers.

The ALBI score and NLR both demonstrated good performance and reliability in predicting outcomes after the Whipple procedure. The use of the ALBI score in predicting mortality has been highlighted in several survival analysis studies. In one investigation, PC-resectable patients with high ALBI grade (>-2.6) and high CA 19-9 (>35 U/mL) exhibited a considerably lower five-year OS rate (13.8%) compared to those with low ALBI grade (<-2.6) and low CA 19-9 (<35 U/mL) (43.3%) [10]. A study by Imamura et al., focusing on a similar patient group, reported a significant difference in median OS time between high and low ALBI score patients [11]. Notably, the five-year OS rate in the high ALBI score group was lower than the low ALBI score group. Moreover, both investigations consistently demonstrated that the length of recurrence-free survival was shorter in patients with high ALBI scores. These studies underscore the potential value of the ALBI score as a predictive marker for mortality outcomes in patients undergoing PC resection. A new marker derived from the ALBI score is the platelet-albumin-bilirubin score (PALBI) score, which includes platelet level. In a group of patients with resectable PC, a high PALBI score was associated with a median OS of 25.2 months, versus 44.4 months in those with a low PALBI score [19].

In the current study, NLR emerged as a robust inflammatory marker, showing optimal performance. With the ease of assessing NLR from routine complete blood count data, healthcare practitioners can readily evaluate patients' conditions, even when NLR levels are within the normal range. A meta-analysis of patients undergoing resections further demonstrated a significant correlation between high NLR and poor survival outcomes (hazard ratio (HR) = 1.2, 95% CI = 1-1.44, p = 0.048) [6]. Additionally, an association between high NLR and low albumin levels was observed. Similarly, in a meta-analysis encompassing 8,252 patients with PC, elevated NLR was associated with reduced survival rates, particularly in patients who received any therapy [7]. Notably, patients who underwent PD had an increased risk of mortality (HR = 1.9, 95% CI = 1.47-2.47, p ≤ 0.0001). The NLR cutoff values in these meta-analyses ranged from two to five [7].

Unlike the previous studies that utilized survival analysis, the current study used logistic regression analysis to identify individuals who experienced mortality during the follow-up period and to assess the performance of prediction models. The overall model showed good discrimination ability, with an AUC of 77%, indicating the model's capability to distinguish between survivors and non-survivors. The sensitivity of the model was also high, at 74%, suggesting its proficiency in identifying patients at risk of mortality. When analyzing the model's performance on training and testing data separately, some differences were observed, potentially attributed to limited sample sizes in each dataset. However, the use of a bagging logistic regression model helped address the impact of a small sample size, leading to improved AUC and sensitivity values for both training and testing data. Notably, both RPD and OPD demonstrated good AUC values, indicating the predictive potential of the model for each surgical category. These findings highlight the importance of the ALBI score, NLR, and sex as accessible and reliable markers for predicting survival outcomes after PD, independent of the surgical approach adopted.

The effect of bilirubin, albumin, and neutrophils on the immune system could potentially explain the predictability capacity of biomarkers such as ALBI and NLR. Bilirubin suppresses CD4+ T cell responses, and CD4+ T cells play a crucial role in activating the antitumor CD8+ cytotoxic T lymphocyte response [20]. High levels of bilirubin induce apoptosis in reactive CD4+ T cells, leading to a reduction in these cells and subsequently increasing the SII index. Moreover, experimental evidence suggests that bilirubin can reduce the inflammatory response in transplanted organs by promoting the accumulation of T-regulatory cells [21]. These regulatory cells can suppress anticancer immunity, thereby promoting tumor development and progression [22].

Furthermore, the inclusion of albumin in the ALBI score is not only reflective of the patient's nutritional status but also indicative of the body's response to inflammation, which can ultimately affect patient survival. The high ALBI group was associated with high scores of systemic inflammatory markers. The immune-modulatory effect of bilirubin can explain these findings. Bilirubin suppressed CD4+ T cell responses at multiple levels. The CD4+ T cells play a critical role in activating the antitumor CD8+ cytotoxic T lymphocyte (CTL) response [23].

In addition to the systemic inflammatory response, NLR mirrors the tumor immune microenvironment, characterized by high infiltration of myeloid cells and low levels of lymphocytes [22]. While the interaction between neutrophils and T cells in inflammation is not yet fully understood, research suggests that a particular neutrophil subpopulation may downregulate CD4+ T cell proliferation and activity [24]. Apart from its prognostic utility, NLR has also been investigated as a predictive factor for the use of immune checkpoint inhibitors [24], highlighting its potential role in guiding treatment decisions in the context of immunotherapy. The interplay between bilirubin, albumin, and neutrophils with the immune system provides a plausible explanation for their value in predicting mortality after pancreatic resection, contributing to a deeper understanding of the factors influencing patient outcomes in this clinical setting.

This study has several strengths. This study used an easily accessible, cost-effective marker that has been tested independently in previous studies. Also, this is the first report combining the ALBI score and NLR to predict the mortality risk of patients with PC and before PD. In addition to predicting mortality, these biomarkers can be used in surveillance to monitor patients with high biomarker levels more frequently. The purpose of this monitoring is to identify signs of disease recurrence or progression after treatment and to provide early therapies to improve patient outcomes. The C-statistics provide a confident model with good discrimination. This study also had some limitations. One limitation was the retrospective nature of the study. Only 60% of the patients had CA 19-9 collected, limiting its use as a pre-surgical marker. Furthermore, the study included a single center with a relatively small sample size, which decreased the statistical power affecting the p-values of other markers. Future consideration is to include a pretreatment marker provided by cross-sectional images. Further studies will be needed to validate the study findings and confirm the applicability of these two markers. A provocative future study could potentially evaluate the impact on survival when these markers are proactively and preoperatively modified by nutrition and decompression interventions.

## Conclusions

The ALBI score and NLR are easy and accessible markers. Their use, combined with the patient’s sex, can provide valuable pre-surgical information to identify those at risk of early mortality after the Whipple procedure. This approach can enhance adherence to neoadjuvant therapy, expedite the early initiation of adjuvant therapy post the Whipple procedure, and increase surveillance for local recurrence or metastatic disease in patients with high pre-surgical biomarkers. In the future, it may also help select patients who would require maintenance chemotherapy after surgical intervention and after adjuvant chemotherapy, improving patients’ outcomes and survival.
